# Testing the Applicability of Nernst-Planck Theory in Ion Channels: Comparisons with Brownian Dynamics Simulations

**DOI:** 10.1371/journal.pone.0021204

**Published:** 2011-06-23

**Authors:** Chen Song, Ben Corry

**Affiliations:** 1 School of Biomedical, Biomolecular and Chemical Sciences, The University of Western Australia, Perth, Australia; 2 Department of Theoretical and Computational Biophysics, Max Planck Institute for Biophysical Chemistry, Göttingen, Germany; German Cancer Research Center, Germany

## Abstract

The macroscopic Nernst-Planck (NP) theory has often been used for predicting ion channel currents in recent years, but the validity of this theory at the microscopic scale has not been tested. In this study we systematically tested the ability of the NP theory to accurately predict channel currents by combining and comparing the results with those of Brownian dynamics (BD) simulations. To thoroughly test the theory in a range of situations, calculations were made in a series of simplified cylindrical channels with radii ranging from 3 to 15 Å, in a more complex ‘catenary’ channel, and in a realistic model of the mechanosensitive channel MscS. The extensive tests indicate that the NP equation is applicable in narrow ion channels provided that accurate concentrations and potentials can be input as the currents obtained from the combination of BD and NP match well with those obtained directly from BD simulations, although some discrepancies are seen when the ion concentrations are not radially uniform. This finding opens a door to utilising the results of microscopic simulations in continuum theory, something that is likely to be useful in the investigation of a range of biophysical and nano-scale applications and should stimulate further studies in this direction.

## Introduction

Biological ion channels are membrane bound proteins responsible for rapidly moving ions across the cell membrane. They play a major role in the transmission of electrical signals within the brain, nervous system and muscles, and their malfunction is associated with a range of diseases [1]. Therefore, understanding them at the molecular level and relating their structure to their function is essential for improving our knowledge about these fundamental components of biology and in finding treatments to ion channel related diseases. One important step in this direction is to be able to predict the ion conductance for a given structure and much research has taken place into finding efficient means of doing this.

Accompanying the rapid progress of experimental techniques, especially driven by the emergence of more and more high resolution structures of ion channels, there have been a lot of efforts to perform theoretical studies on the ion channels because such studies can provide experimentally unaccessible insights. For example, molecular dynamics (MD) simulations have been widely used to give atomic level insight into the function of channels, such as the steps involved in ion conduction [2], possible gating mechanisms [3]–[7] and how selective transport can arise in these pores [8]–[10]. MD has even been used to simulate ion conduction with an external electric field up to a microsecond timescale [11]–[14]. However, directly predicting the channel conductance using MD is very computationally demanding which makes calculating statistically meaningful values of ion conductance unreachable for most investigations.

Brownian dynamics (BD) simulations provide an alternative method for predicting the conductance of a given structure [15]–[21]. In these, only some atoms (usually the ions) are simulated explicitly, moving in a stochastic manner under the influence of random and frictional forces in addition to electrostatic or average forces arising from other ions and the protein. By adopting approximations such as considering the protein and water as continuous dielectric media, BD can be easily used to simulate the motion of ions on the microsecond timescale. Therefore, many ion conduction events can be observed and statistically meaningful conductances can be determined. But, such approximations also have drawbacks. For example protein motions and fluctuations are usually ignored, and highly detailed atomic interactions such as that between the ions and water are mostly unaccounted for.

Continuum theories provide another computationally efficient method for calculating channel currents. In these ionic flux is generally determined from the Nernst-Planck (NP) equation (drift-diffusion) that was well established for bulk electrolytes. While the NP equation has long been applied to studying ion channels [22], [23] it requires prior knowledge of the electrostatic potential and ion concentrations as well as extension to multi-ion permeation [24]. The most common way of overcoming this is to combine the NP equation directly with Poisson's equation yielding the so called Poisson-Nernst-Planck (PNP) theory [25], which has also been widely used in the last two decades [26]–[30]. The use of PNP theory in ion channels was motivated by macroscopic ion transport studies wherein the ions are also considered as continuous charge distributions. By using PNP, one can calculate the ion concentration, electrostatic potential, and ion flux in a single short calculation on a desktop computer. Therefore, the continuum approaches require much less simulation time than microscopic approaches such as MD. However, previous work by Corry et al. has shown that the simple implementation of PNP is flawed at the microscopic scale due to the over simplistic representation of the few ions in the channels by their mean field properties, and particularly by the overestimation of the shielding of forces on permeating ions by counter ions [31]. Although there has been some effort to improve PNP theory by introducing additional terms to the PNP equations or using explicit ions in the calculation [32]–[35], the results are still not satisfying and the number of open parameters make it less attractive if the aim is to determine the likely conductance of a given structure. There are several good reviews about the use of MD, BD and PNP methods for studying ion channels which are recommended for further reading [36]–[38].

Since the main reason for the failure of the PNP theory in ions channels is the incorrect prediction of ion concentration in narrow pores [31], it is worth investigating whether the Nernst-Planck theory can still be used if the ion concentrations can be determined in a more reliable manner. Is the Nernst-Planck theory when used alone applicable for use in narrow ion channels if the ion concentration and the potential could be correctly obtained? If so, then alternative approaches for determining channel conductances may be possible that can balance computational cost and accuracy. Indeed, there has been some pioneering work in this direction, such as the calculation of ion concentration by using Monte Carlo (MC) or density functional theory [39], [40], and the use of ion concentrations obtained from MD or Monte Carlo (MC) methods directly within the NP equation [41]–[43]. The motivation of this kind of combination is that it is hoped that shorter simulation times are required to estimate the ion concentration and diffusion coefficient (which can then be used in the NP equation) than would be required to directly predict ion currents. For example, Allen et al. used molecular dynamics simulations and the umbrella sampling method to calculate the potential of mean force (PMF) and ion concentration in the gramicidin channel, and then used the NP equation to estimate the maximum conductance of the channel, something that took less computational effort than directly simulating the ion current [43]. However, despite its use in this context, the primary mystery of whether the NP theory is valid in the microscopic world remains unresolved. This is an essential problem that must be solved before further effort in this direction are carried out.

Therefore, we aimed to test if NP theory is applicable in narrow ion channels by combining it with BD simulations. That is, we determined the time averaged ion concentration and electrostatic potential in the channel directly from BD and used these as input to perform NP calculations from which we determined the channel current. The reason for conducting the calculation in this way is that the current can also be determined directly from the BD simulations. Thus, the NP and BD simulations will be utilising consistent concentrations and potential, but determining the conductance in two different ways. In this way we can directly check if the current obtained from the continuum calculation is the same as that found using explicit simulations of the ions. While BD simulations have been shown to be able to reliably predict channel currents in a number of cases, this is not critical to the present study. Rather, we aim to see if the continuum approach can provide results in accord with that found when employing explicit ions. To test if the NP equation is valid in various situations, we performed our tests in a series of sequentially more complex channel models: cylindrical channels without dielectric boundaries, cylindrical channels with dielectric boundaries, cylindrical channels with dielectric boundaries and fixed charges in the channel wall, non cylindrical channels and a realistic model of the transmembrane (TM) domain of the mechanosensitive channel of small conductance (MscS) derived from a recently determined crystal structure [44]. The aim of using these different channel models is to examine if the accuracy of the NP equation is influenced by the channel radius, the channel shape, the channel occupancy, the rate of change of ion concentrations or forces in the pore, or differences in the cation and anion concentrations. In the results section we show that general agreement between the two approaches is found in all situations although discrepancies arise when the concentration of one ion is much lower than the other, before we discuss the potential applications and limitations of the proposed method of calculating channel currents.

## Methods

### Nernst-Planck theory

The NP electrodiffusion equation is widely used in the continuum theory of non-equilibrium processes such as ion transport, and can be written as follows:
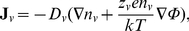
(1)where 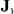 is the flux of each ion species, 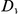, 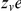, and 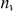 are diffusion coefficient, charge, and number density of the ions of species 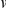, respectively. 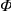 is the electrostatic potential (ESP) in this case. In our 1D case, it can be written as:
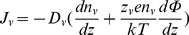
(2)


To evaluate the ion fluxes, there are three main parameters or variables that need to be determined. The first is the diffusion coefficient of each ion species. Many previous studies keep this variable as an open parameter that can be adjusted to fit the experimentally determined conductance values, but this approach is not satisfying if the aim of the study is to determine the likely conductance of a given channel structure. In some other cases the values of the diffusion coefficients have been determined directly from MD simulations which show this to be position dependent [19], [45]–[49]. In general, the value usually decreases by 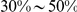 in the interior of the channel compared to that in bulk water. But in some studies, a value lower than 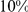 of the bulk value was obtained [6], [50], which leaves the determination of the diffusion coefficient rather uncertain and highly system dependent. The second variable is the number density 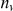 (in SI units), which is related to the ion concentration 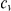 (in moles/liter) through 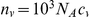. Finally, the third variable is the ESP, 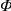. In the most widely used version of PNP theory, the ion concentration and ESP are obtained by simultaneously numerically solving Poisson's equation and stationary NP equation iteratively [27]. But, as noted previously, the mean field approximation implicit in this encounters problems in narrow channels [31]. Alternative approaches have used MD [43] or MC [41], [42] methods to get the ion concentration for input to the NP equation, but none of these studies had a clear way to tell if the combination of these methods is reliable.

### Brownian dynamics

BD simulations have been successfully applied to determine channel currents and ion conduction events in various ion channels in recent years [18], [19], [51]–[54]. In BD, the motion of individual ions is traced explicitly, but the water and protein atoms are treated as continuous dielectric media [17], [18]. In these simulations the channel is usually taken to be a rigid structure during the simulation (see [55] for an exception), and partial charges are assigned to the protein based upon the atomic positions. Ions are given starting positions in or around the channel and the motion of these ions under the influence of electric and random forces is then traced using the Langevin equation.

In the present case, most of the channel models were made from idealised shapes and a small number of partial charges were added at specific positions described in the results section. The one exception was for the studies of the MscS channel in which the pore was centred on the 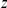-axis and a smooth water-protein boundary of the channel was defined by rolling a 1.4 Å sphere representing the water molecule along the surface. The boundary was symmetrised by taking only the minimum radius at each 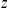-coordinate, and then the curve was rotated by 360 to obtain a three-dimensional channel structure with radial symmetry. In this case partial charges were assigned using the CHARMM27 all atom parameter set [56].

In all cases, 16 pairs of Na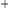 and Cl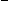 were randomly distributed in 30 Å reservoirs that mimic the intra- and extra-cellular solution to bring the ion concentration to 300 mM. A time step of 100 fs was used and the trajectory was saved every 100 steps. Electrostatic forces were precalculated by assigning dielectric constants to the protein, channel interior and bulk water and solving Poisson's equation using an iterative method [57] and stored in tables to speed up the simulation [58]. While the dielectric constant in the channel is uncertain, we follow previous studies that have shown the best results in channels of this dimension are obtained assuming dielectric constants of 2 for the protein and 60 for the channel interior [54], [59]–[62]. While the dielectric constant of the bulk water is likely to be closer to 80, for computational ease it is also set to 60 and the Born energy barrier for the ion to move between the dielectric constants of 80 and 60 is included as an additional force as in the previous studies above. We note that the exact choice is not critical for this study provided a consistent set of parameters is used in both the BD and NP calculations. The current is determined directly from the number of ions passing through the channel. In all cases described here an electric field of 20 mV/nm was applied to create a membrane potential along the z direction by incorporating the electric field into the solution of Poisson's equation, rather than simply applying forces on the ions. The boundaries between channel and water was treated as rigid walls from which ions elastically scatter, i.e., when the ions get to the channel boundary as close as 0.55 Å, the radial velocities of the ions would be multiplied by −1 while the axial velocities keep unchanged. The ions thus only move in the water environment and the ion-ion interaction can be calculated from Coulomb's law with an additional short range potential that reproduces the ion-ion radial distribution function found in all atom MD simulations [51]. All BD simulations were run for 1.6 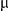
s. More details about the BD simulation methodology can be found in previous studies [17], [18], [52].

### The combination of Brownian dynamics with Nernst-Planck theory

In order to test the validity of the NP equation, we incorporated the ion concentrations and potential found from BD simulations into the NP equation (BD-NP) to determine the channel current for comparison with those found directly from BD. Thus, a method of combining the results of BD with the NP equation needed to be determined for this study. As mentioned above, three quantities are needed for NP calculations: the diffusion coefficient, ion concentration and potential. In our tests we derive each of these directly from the corresponding BD simulations.

Since we only need to make sure that the same diffusion coefficients are used for both the BD and BD-NP methods, we can choose any arbitrary value for this as it should not affect our final comparison. To make things simpler, we adopted the diffusion coefficients of ions in bulk water for both BD and BD-NP calculations, which are 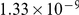 m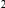/s for Na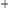 and 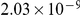 m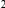/s for Cl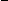 respectively.

The ion concentrations are calculated from the BD trajectories. For each channel model, a 1.6-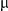
s BD simulation was performed. The first 0.2 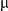
s was assigned as equilibration and not considered for data analysis. The latter 1.4-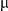
s BD simulation trajectory was utilised to calculate the one dimensional (1D) ion concentration with a grid spacing of 0.5 Å, which was then implemented to NP equation for further calculation. Please refer to the supporting information to find more details about this (Text S1 section S1.1, and figure S1).

To make sure that the electrostatic potential determined from the BD simulations is consistent with the ion concentration, we proceeded in two steps. First we fixed the value at the end points of the calculation region to be that found from solving Poisson's equation (as done for calculating the force in BD). Next we determined the values in between by solving the stationary NP equations which enforces that the flux though the channel is the same at all points along its length:
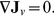
(3)Further details of the implementation of this strategy can be found in Text S1, section S1.2. The ESP could be determined in other ways, for example by solving Poisson's equation at each snapshot of the BD trajectory and averaging, but the approach described above is less sensitive to slight fluctuations in the average potential which are amplified when calculating the flux (please cf Text S1 section S3, figure S3 and figure S4).

The diffusion coefficients of ions, the ion concentrations and the potentials determined from the BD simulations were put into the NP equation 2 to calculate the currents as described in the Text S1, section S1. In all these calculations, we adopted a grid spacing of 0.5 Å which gives the most stable prediction of ion currents (please cf table S1).

## Results and Discussion

### BD vs BD-NP for passive cylindrical channels

**Figure 1:**
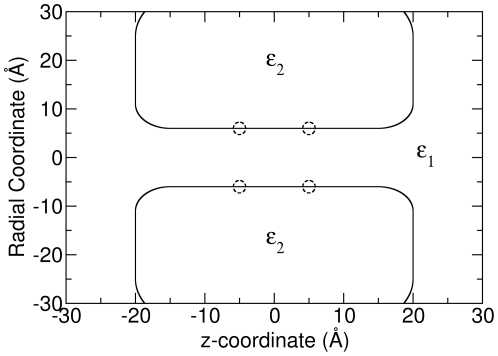
The cylindrical channel model. A 6-Å-radius model is shown here. The dashed circles show the positions of the charged rings in the charged cylindrical channel.

We started our test with the simplest model — a cylindrical channel as shown in figure 1 with no dielectric boundaries (in this case a dielectric constant of 60.0 is used throughout). We term this a ‘passive’ channel to reflect the fact that there are no induced forces on ions from the channel walls and ions simply elastically scatter from the water/channel interface. The channel has a cylindrical shape spanning from 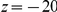 Å to 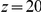 Å with the central axis of the channel aligned on the z axis. A series of such models were built with the radii of the channels ranging from 3 Å to 15 Å. In the NP calculations, values of the ion concentration and potential in the segment between −15 Å
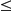
z
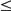
15 Å was considered, although the choice of the segment was found not to influence the results provided we avoided including the reservoirs where the channel shape changes rapidly.

**Figure 2:**
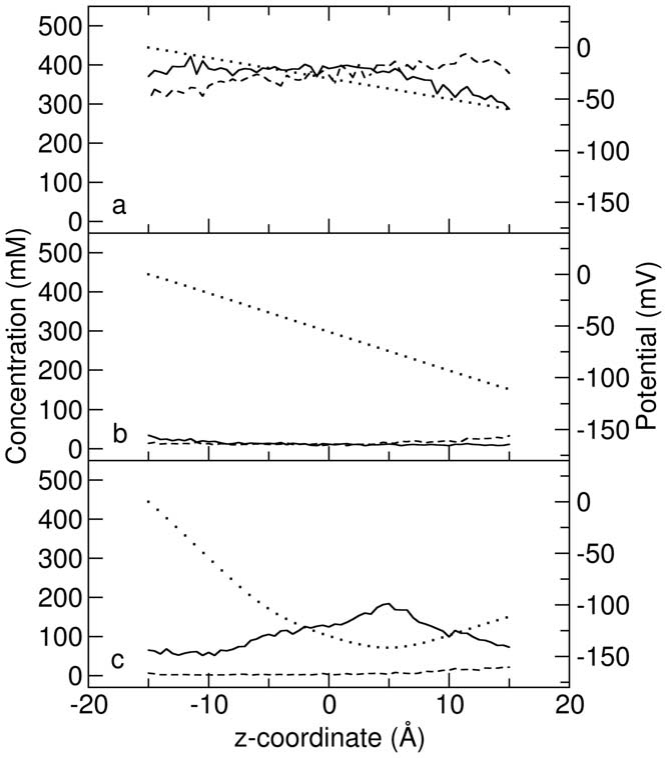
The ion concentration and ESP of the (a) passive, (b) real and (c) charged cylindrical channels with a radius of 6 Å. The concentration of Na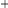 and Cl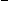 are shown with solid and dashed lines respectively, and the ESP is shown with the dotted line. The ESP shown is that obtained in the absense of mobile charges.

**Figure 3:**
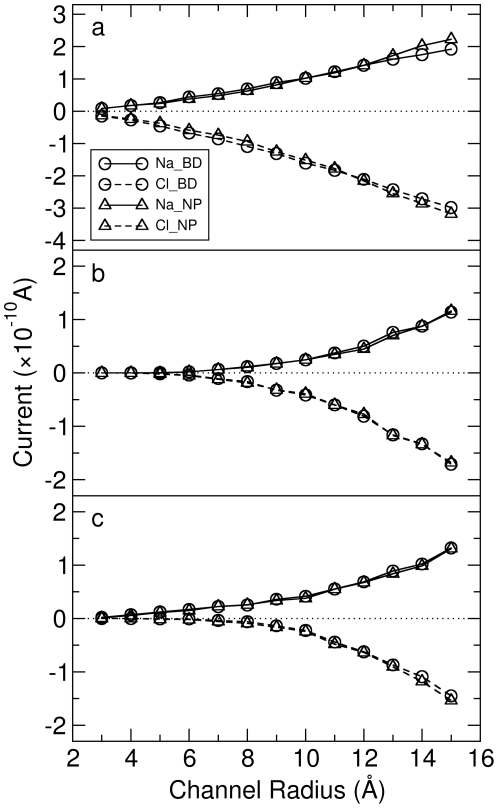
The currents of Na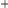 and Cl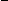 through the (a) passive, (b) real and (c) charged cylindrical channels of differing radius under 20 mV/nm electric field found using BD simulations and the BD-NP method. The error bars are smaller than the size of the symbol and therefore not shown here.

An example of ESP in a passive cylindrical channel with radius of 6.0 Å is shown in figure 2a with the dotted line. Our method of calculating the ESP accounts for not only the external applied electric field, but also the dielectric boundary and fixed charges in the system. But, in this case, since there is neither a dielectric boundary nor charge for the passive channel, the potential changes linearly through the pore. Meanwhile, [Na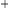] and [Cl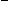] are also shown in figure 2a, as calculated from the last 1.4 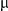
s BD trajectory. The concentrations are fairly flat in the channel, however, [Na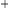] shows a slight decrease and [Cl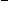] a slight increase along the direction of the electric field caused by the build up of concentration on the membrane surface around the ends of the channel. The current carried by Na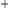 and Cl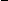 found using each method is shown in figure 3a. As can be seen, the BD-NP results match pretty well with the BD results at all the channel radii studied. Even in the narrow channels the current is reproduced with a high degree of accuracy indicating that the concept of combining BD and NP in this way to determine the channel current is reasonable. The agreement in this case is not surprising given that the PNP theory also predicts accurate currents at all radii in these passive channels [31].

### BD vs BD-NP for real cylindrical channels

To make further tests in more realistic channels, we utilized ‘real’ cylindrical channels for which the dielectric constant of water 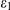 was set to 60.0, while the dielectric constant of the channel 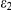 was set to 2.0 as shown in figure 1. This means that the channel body is now more distinct from the water and there will be induced surface charges on the channel boundary in the presence of ions. As there are no permanent charges in this case (as would arise from partial charges on the protein atoms) we are able to study the effect of the dielectric boundary in isolation. All the other parameters were the same as those for the passive cylindrical channels.

The ESP and ion concentration from the BD trajectory for a real cylindrical channel with radius 6 Å are shown in figure 2b. The ESP also decreases linearly like in the passive cylindrical channels because there are no point charges on the channel, but in this case there is a larger potential drop due to the existence of the dielectric boundary. The ion concentrations show very low values in the channel interior, exhibiting a distinct difference from those in passive channels. This is expected as in this case, the low value of the dielectric constant in the protein leads to induced surface charges on the dielectric boundary that have the same sign as the conducting ions and repel the ions from the channel wall, effectively creating a dehydration barrier for ions to enter the pore.

The ion currents calculated from BD and BD-NP methods are shown in figure 3b. Although the currents are lower than in the corresponding passive channels, the results from the two different methods still match well. This is a significant finding, especially when recalling that the PNP theory completely fails in the narrow channels used here [31] his reinforces the fact that the failure of PNP in narrow channels originates from the incorrect prediction of ion concentration calculated by the combination of Poisson's equation and the NP equation. If the ion concentration can be obtained from more accurate method, such as BD simulations here, then the NP theory is able to accurately predict the current for these channels.

### BD vs BD-NP for charged cylindrical channels

So far we have considered fairly simple channel models in which the ion concentration and potential vary smoothly throughout the pore and in which the channel is either passive, wide, or narrow but containing very few ions. In most realistic cases none of these conditions will hold and it is important to check if more rapid fluctuations in ion concentration, ESP or multiple occupancy influence the accuracy of the NP results. For example, in the classical model, the atoms in proteins carry partial charges, and often the presence of charged rings or functional groups at specific positions near the pore is used to control ion permeation and select between different ion types. The presence of such charges can create more rapid changes in the ion concentration, ESP as well as multiple occupancy.

To mimic this effect and study how the BD-NP method behaves under this more complex situation, we built ‘charged’ cylindrical channels. All the parameters for these charged channels are the same as those for the real cylindrical channels, except that there are two charged rings in the channel. As shown in figure 1, the dashed circles at 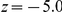 and 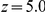 show the positions where 16 point charges were manually fixed at the channel boundary. At each position, 8 point charges each with a charge of −0.09 e were uniformly distributed at the channel boundary. Therefore, each of the two rings has a net charge of −0.72 e, which is expected to make it easier for cations to enter the channel than anions [63]. These point charges were treated statically to mimic charged atoms, as often seen in ion channels, rather than intending to represent the dielectric polarization.

The ESP from electrostatic calculations for a 6-Å-radius charged cylindrical channel is shown in figure 2c. It is obvious that there is a potential well located at around 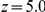 due to the combined effect of the two charged rings and the membrane potential. Correspondingly, [Na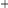] has a maxima at this position due to the electrostatic interactions with the charged rings. In contrast, [Cl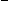] remains at very low values throughout the channel. The charged rings do act to form a selectivity filter by attracting more cations into the channel and repelling anions.

The ion currents for all the charged cylindrical channels are shown in figure 3c. Again, the BD-NP results generally match well with those from BD simulations. Furthermore, the negatively charged channels do have cation selectivity, which is especially obvious when the channel radius is small. This is very encouraging which means that the BD-NP method is applicable to all the cylindrical channels, even if the channels are narrow, charged and selective or if there are non-monotonic ion concentrations and electrostatic potentials. One thing to mention here is that when the channel is very narrow (radius 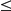
7 Å) and negatively charged, the current of the Cl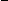 is less accurate. This is not obvious in figure 3c because those values are 1
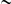
2 orders less than those of Na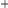. We will discuss the importance of this later in the paper.

We also tested whether the exact value of the dielectric constant influences the reliability of the BD-NP method. To this end we have repeated all the tests for the passive, real and charged cylindrical channels with water dielectric constant set to be 80.0, and the results are found to be as good as those described above (shown in figure S2). Therefore, we believe that the BD-NP method is capable of predicting ion fluxes and currents as well as BD simulations themselves in cylindrical channels, irrespective of the channel radius and the choice of dielectric constant.

### BD vs BD-NP for more complex ‘catenary’ channels

**Figure 4:**
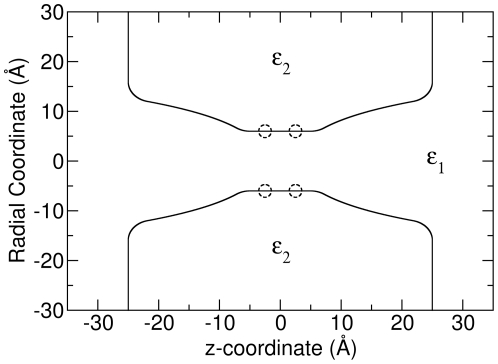
The catenary channel model. The dashed circles show the positions of the charged rings.

It is possible that the success of the 1D BD-NP approach lies in part due to the simple cylindrical shapes being employed and any deviation from such simple shapes is more likely to stress the 1D calculation. To examine if BD-NP works for channels with more complex shapes, we did further tests on a ‘catenary’ channel model. The channel structure is shown in figure 4. The middle part of the channel (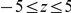) is a cylinder which has a radius of 6 Å, while the outer parts of the channel (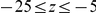 and 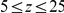) has a catenary shape with the radius changing from 6 to 12 Å. Similar to the study on the cylindrical channels, tests were made on a passive, real and charged catenary channel. For the passive catenary channel, the dielectric constants for water and channel were both set to 60.00; for the real catenary channel, the dielectric constants were set to 60.00 and 2.00 respectively; for the charged catenary channel, the dielectric constants were the same as for the real channels, plus two negatively charged rings were put on the channel boundary as shown with dashed circles in figure 4. Each ring has 8 uniformly distributed point charges with the value −0.045 e, resulting a total charge of −0.36 e per ring.

**Figure 5:**
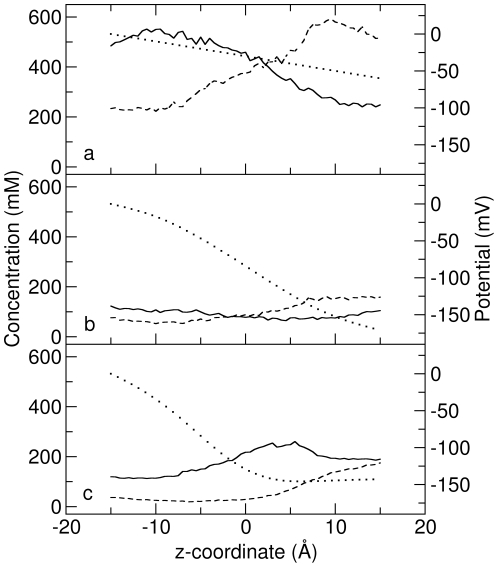
The ion concentration and ESP of the (a) passive, (b) real and (c) charged catenary channels. The concentration of Na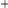 and Cl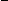 are shown with solid and dashed lines respectively, and the ESP is shown with the dotted line.

The potential and ion concentration for each catenary channel, calculated from the BD simulation, are shown in figure 5. We can see that these profiles share similar features to those for cylindrical channels except that the ion concentration at the outer parts of the channel is higher because ions can build up on the narrowing faces of the pore entrances. Also, the ion concentration in the real catenary channel is much lower than in the passive channel due to the induced surface charges at the boundary. In the negatively charged catenary channel, the ion concentration of Na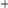 is much higher than Cl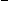 due to the electrostatic interaction, especially in the middle narrow part of the pore.

The currents determined with the BD and BD-NP methods are shown in table 1. We can see that the BD-NP method still works well in general. The biggest difference arises for Cl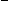 in the charged catenary channel where the BD-NP calculation gives a value about 70% higher than the BD result which is discussed in more detail below. Apart from this, the BD-NP method seems not affected by the shape and the change of the radius of the channel, which means NP could be valid in more generic channels with complex shapes. Additional tests with wider radius and different charges at the boundary were also performed, which showed that the BD-NP method works better in wider charged catenary channels and the amount of the charges can affect the accuracy of the results. All the BD-NP results presented above used the central segment of the channel 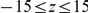 in the calculations and thus included the region where the pore radius is changing. The choice of calculation region was not found to be important to our results as those found using the regions 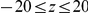 or 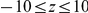, are almost identical to those presented above. The only time that the results differed was when we included the ends of the channel and the sharp radius increase at the start of the reservoirs that occurs at 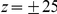. The fact that the BD-NP method works well in the situation where the channel radius is not constant is very encouraging considering the fact that we are doing 1D BD-NP calculations. The additional tests results are shown in table S2.

**Table 1:** Currents through the catenary channels (A).

	passive		real		charged	
	Na 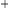	Cl 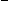	Na 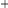	Cl 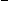	Na 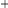	Cl 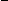
BD	7.43 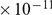	−1.01 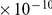	2.39 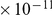	−3.89 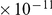	4.94 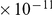	−1.26 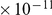
BD-NP	6.38 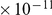	−9.45 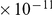	2.56 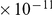	−4.55 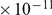	4.83 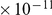	−2.13 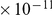

### BD vs BD-NP for the transmembrane domain of MscS

**Figure 6:**
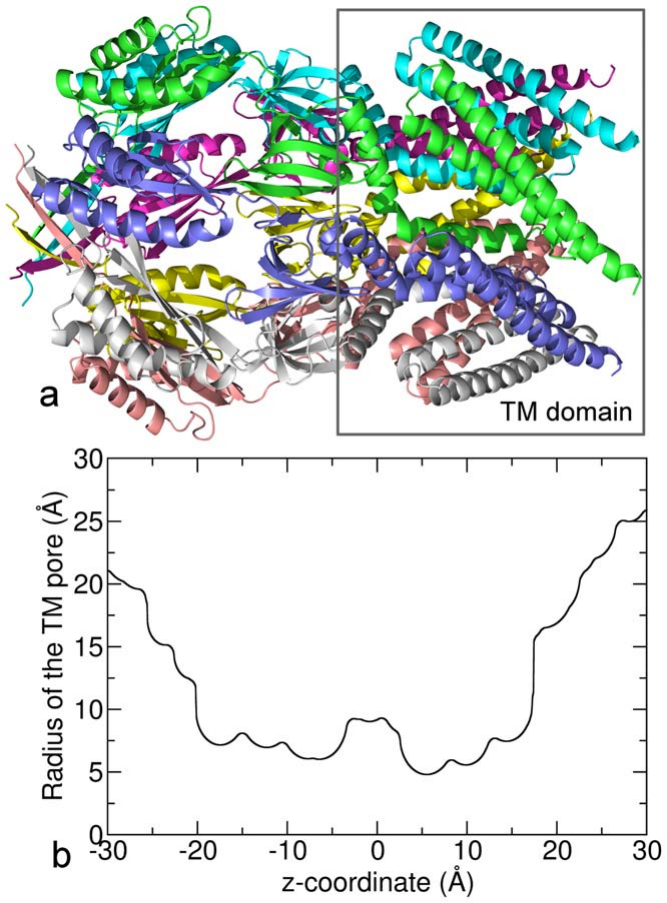
The model of the mechanosensitive channel MscS. (a) The structure of MscS with the TM domain marked with the rectangular box. (b) The radius of the TM domain of MscS.

Finally we tested the BD-NP method for a more realistic channel model — the TM domain of MscS — as a first step to practical applications. MscS is one kind of mechanosensitive channel that opens in response to mechanical forces in the lipid bilayer. In this work, we only took the TM domain of the protein (PDB entry 2vv5 [44]) as illustrated in figure 6a and performed 1.6 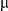
s BD simulation on it. The radius of the channel is shown in figure 6b and is complex in shape and the channel is highly charged with a total charge of 35 e, which provides an ideal model to test under a very complex realistic situation including large concentrations and thus multiple ion occupancy.

**Figure 7:**
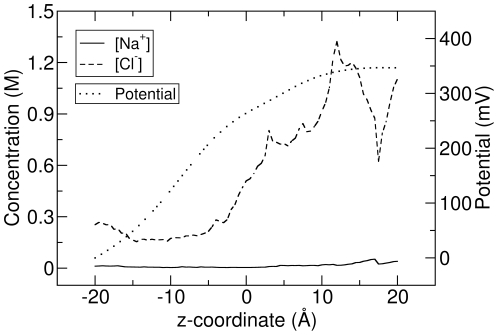
The ion concentration and ESP in the TM domain of MscS. The concentration of Na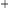 and Cl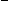 are shown with solid and dashed lines respectively, and the ESP is shown with the dotted line.

The ESP and ion concentration from BD simulation are shown in figure 7. There is a large potential difference across the chosen segment (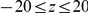), about 350 mV. The concentration of Cl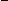 is much higher than Na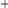 and even much higher than the bulk concentration 300 mM in some particular locations of the channel interior (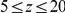) due to the high positive charges on the protein. The ion currents from BD simulations are 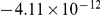 and 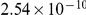 for Na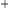 and Cl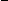 respectively, showing an anion selectivity of the TM domain. The ion current from BD-NP calculation are 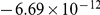 and 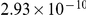 for Na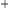 and Cl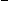 respectively. Therefore, the BD-NP method overestimates the current about 63% for Na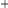 and 15% for Cl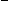 when comparing to the BD simulation results.

### The effects of shape and charge

From the above results, we can see a trend: the BD-NP method becomes less accurate when increasing the complexibility of the channel. Two factors might be responsible for this: the shape of the channel and the charge distribution on the channel. Exploring to what extent the two factors affect the accuracy may direct us to the way to improve the method.

To see how the shape of the channel affects the accuracy of the BD-NP method, we can first compare the ‘passive’ channels without dielectric boundaries or charge distributions. For all the passive cylindrical channels, the results of BD-NP match well with BD as shown in figure 3a, which means the radius of the channel is not a key factor that influence the accuracy of the results. When changing the shape to the ‘catenary’ channel, the results from BD-NP and BD alone still match well as shown in table 1. To further verify this point, we ran an additional BD simulation on a ‘passive’ TM domain of MscS, i.e., the shape of TM domain of MscS (as shown in figure 6b) was utilized to generate a channel without any dielectric boundary or charge. In this simulation, the Na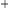 currents calculated from BD and BD-NP are 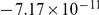 and 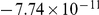 A, and the Cl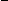 currents calculated from BD and BD-NP are 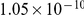 and 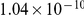 A respectively. The result indicates that even in a very complex shape like in a real ion channel, the results from the two methods are very close. Therefore, we believe that the shape of the channel does not have a major influence of the accuracy of the BD-NP method.

As mentioned above, when the cylindrical channel is narrow and charged, the current predicted by the NP equation for the ion of lower concentration is less accurate. This is a sign that the charges on the channel might be affecting the accuracy of the NP calculation. To further study this effect, we can examine the results of the catenary and MscS channel. For the catenary channel, when changing the channel from passive to charged, the accuracy clearly decreased especially for the Cl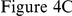 which is the minority ion type as shown in table 1. For the MscS TM domain, we can see similar trend in table 2. It seems that the charge distribution on the channel does have a clear influence on the accuracy of the BD-NP method. To further understand this, we examined how the current passing through the 6-Å-radius cylindrical channel changed as we slowly increase the charge on the pore wall. As shown in table 3, we can see that as the charge on the channel increases from −0.36 e per ring to −2.88 e per ring, the deviation in the Na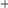 current predicted from NP compared to BD increases from −3.35% to 35.36%. Interestingly, the current of the minority ion type Cl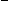 are very different from the BD results, however, the absolute magnitude of the Cl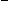 is also 1
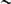
2 orders smaller than for Na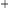. When the charges on the channel is −5.76 e per ring, the NP equation does not have a solution for the Cl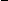 current any more, though the deviation for the Na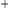 current is −25.85% and still in a reasonable range.

**Table 2:** Currents through the TM domain of MscS (A).

	passive		real		charged	
	Na 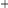	Cl 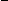	Na 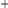	Cl 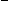	Na 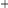	Cl 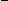
BD	−7.17 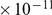	1.05 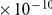	−4.23 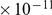	6.51 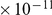	−4.11 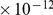	2.54 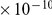
BD-NP	−7.74 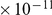	1.04 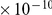	−5.90 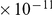	8.33 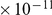	−9.48 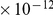	4.63 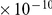

**Table 3:** Currents through the charged 6-Å-radius cylindrical channels (A).

Charge per ring (e)	−0.36		−0.72		−1.44		−2.88		−5.76	
Ion type	Na 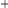	Cl 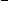	Na 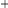	Cl 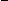	Na 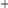	Cl 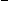	Na 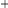	Cl 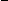	Na 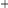	Cl 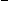
BD	6.17 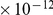	−8.00 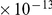	1.70 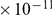	−1.03 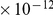	2.51 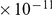	−8.00 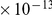	3.30 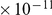	−4.57 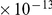	3.69 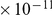	−3.43 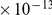
BD-NP	5.96 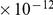	−2.51 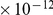	1.48 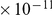	−2.00 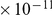	2.84 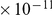	−5.56 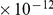	4.47 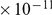	−6.49 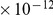	2.74 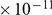	NA
Dev	−3.35%	213.63%	−12.76%	94.47%	13.19%	595.50%	35.36%	1320.57%	−25.85%	NA

‘NA’ means there is no solution for the stationary NP equation for this case.

The above analysis shows that the charge distribution on the channel has a much greater affect on the accuracy of the NP results than the shape of channel, but the reasons for this deviation are yet to be established. The most likely reason for the inaccuracy is that the presence of permanent charges creates a non-uniform ion distribution in the channel. We adopted a 1D approximation in the NP calculations, and it can be expected that a smooth, uniform ion distribution would give the best results. But, if the channel has a negatively charged ring, for example, then there would be a high cation distribution and low anion concentration near the channel boundary. The 1D NP calculation does not capture this and only uses the average concentration at any position along the channel. We believe that this difference is the key factor that causes the deviation between the BD and NP currents.

Although there are obvious discrepancies between the BD and NP results, we still believe the NP equation is applicable for estimating currents in the majority of cases. Firstly, although the percentage difference is greatest for the minority ion types, the total current is mostly dictated by the majority ion whose conductance is predicted more accurately. Secondly, these results are much more accurate than equivalent PNP results. Finally, the ability to predict the current to within 30% in the worst case scenarios still allows for the qualities such as the conductance state of the channel to be determined. Although PNP can also be used to estimate the conductance of a wide channel, it becomes less reliable for narrow ones [31]. The BD-NP method appears to have a greater range of validity, being able to estimate the magnitude of currents in both narrow and wide pores. If one extends the NP calculation to 3D, the accuracy could probably be enhanced, but this is beyond the scope of the present study.

### Success, limitation and perspective

We have examined a range of different ion channel models to test the validity of the BD-NP approach including passive, real and charged cylindrical channels with various radii and more complex channel shapes to explore under what conditions the NP theory is still applicable. Although there are some deviations, the BD-NP and BD results show overall good agreement. Results are especially good when the model channel conducts ions with currents larger than 10 pA suggesting that the NP theory can be used to obtain estimates of channel currents provided that the ion concentration can be precisely obtained beforehand.

It is important to consider the reasons for both the similarities of the currents found from BD and those found using BD-NP as well as the differences. In BD, the forces acting on each ion are determined at each timestep in the simulation based upon the positions of all the ions in the system at that time. In contrast, the NP equation was derived from macroscopic Smoluchowski equation in which the average force is calculated in a mean-field manner. In the case of BD, the current is calculated using the instantaneous forces on explicit ions, while in NP current is determined from the time averaged concentration and potential. Thus, although the mean properties found in the BD simulations are consistent (indeed the same) as those employed in the NP calculation, one need not expect identical results. Furthermore, additional differences can be expected to arise since the NP calculations in this work were performed under one dimensional approximation, while the BD simulations were three dimensional. It is not surprising, therefore, that there are some deviations between the results from the two methods. On the contrary, it is quite surprising to see such good general agreement suggesting that the mean field approach is capturing the important physics in most cases. The two cases in which the worst results were obtained using BD-NP suggest some possible limitations in the mean field approach. In both the charged catenary channel and the MscS TM domain, where the concentration of one ion type is extremely low while the other is large, the current of the minority ion type is overestimated by BD-NP, most likely as a result of non-uniform distribution of this type of ions in the channel. We also want to point out that this deviation is not due to insufficient sampling of the ion concentration, as extending the BD simulation three times longer for the charged catenary channel gave no improvement. Therefore, the ion distribution can give an indication of cases where potential errors may arise.

Having noted the conditions when the worst results were obtained, it is worth pointing out that in the majority of cases studied BD-NP can usually give good estimation about ion conductance, with an error below 30% comparing to the BD results (below 10% in most cases). Even in the worst cases, the current of the ion with large concentration and conductance were estimated to within this same level. The general agreement between the BD-NP results and those from BD alone implies the validity of NP in microscopic scale, and that it is possible to use mean-field approximations to study ion channel currents provided that the ion concentration is accurately obtained. However, when the channel is highly charged, the accuracy of the 1D approach decreases. The results from the BD-NP method are clearly better than analogous PNP results which overestimate the currents in channels with dielectric boundaries [31]. Although PNP performed best in charged channels, even in these cases the BD-NP approach appears more accurate. More recent PNP studies have attempted to include dielectric self energy to improve the predicted currents [32]–[35], but this improvement primarily occurs by better predicting ion concentrations. By utilising accurately determined concentrations directly in the calculation, the BD-NP method can be expected to outperform even the modified PNP methods.

One should also keep it in mind that our NP calculations have all been conducted in 1D - that is only average concentrations and potentials in the axial direction of the channel were considered (see SI section S1 for more details). This makes the success of the method even more surprising and suggests that it could be further improved by extension to 3 dimensions. This also explains why it is that when the ion distribution is more uniform and the channel shape is more smooth, the agreement of the two methods is better. One of the main reasons we did not do the 3D calculation here is that accurate results require the concentration to be well sampled at all points in the channel. In 3D this generally requires longer simulations. It should also be noted that the NP calculation is extremely efficient. Once the ion concentration, the potential difference and the diffusion coefficient are known, it takes less than a minute to get the ion current on a single PC.

There are also some limitations in our proposed method for combining BD with NP. The most significant one is that the potentials calculated from [Na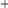] and [Cl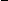] do not match exactly, which is a compromise to make sure we can get constant ion current values through the channel. More results and discussion about this can be found in Text S1, section S3. Another limitation is that the segment chosen to do the NP calculation must be part of the channel (not including the bulk) in order to avoid sudden changes of radius, which is a shortcoming resulting from the 1D approximation. But fortunately, the specific choice of the segment does not matter as long as it is part of the channel proper.

The combination of NP and BD itself is not very exciting as the BD simulations themselves are already able to yield ion currents. Indeed, by the time the concentration is well determined from the BD simulation we already have a statistically reliable conductance value. Thus, there is no need to resort to the more approximate BD-NP method at this stage. Our purpose in doing these calculations was not to propose BD-NP itself as a useful approach to calculating channel currents, but rather to test if NP theory is still valid in narrow ion channels. By making our comparison of the BD-NP results to those from BD alone we can compare currents determined from exactly the same underlying concentration and potential data, that is we know what current we should expect to get from the NP calculation and we can directly test the mean field approximation.

Our encouraging finding is that the NP theory appears to be applicable at the microscopic scale, and our study presents a good example about how microscopic simulations can be related to continuum theory calculations. This method can be easily extended to 3D version as long as ion concentration could be obtained in a more efficient way. We believe that this direction could be further pursued to find a more useful way of getting reliable channel conductances from detailed microscopic simulations. For example, one might want to try combining other methods for determining the ion concentrations in the channel (but that cannot themselves reliably predict channel currents) with NP. The first natural consideration is MD. Predicting channel currents is difficult in MD due to restrictions on the timestep and the computational power required. However, MD can explicitly account for the interactions between water molecules, and can more easily account for protein flexibility than BD, both of which may be important considerations in determining ion permeation. In principle, the ion concentration, diffusion coefficient and electrostatic potential could all be obtained from MD simulations, which could be taken as inputs for further NP calculations to predict the ion conductance. This would be useful if it could be done using shorter simulations than those needed to directly simulate multiple conduction events in the MD simulations themselves. The trickiest problem to overcome, however, is to work out how to reliably calculate the ion concentration as it can be hard to get sufficient sampling of ions in the channel using MD. To do this, some advanced simulation techniques such as umbrella sampling might be needed to get more statistically meaningful values, ideally to produce a potential of mean force (PMF) that can be employed in the NP calculation, an approach that has been tried previously by Allen et al. in gramicidin [43], [64]. By combining these PMFs with the NP equation, our results show that it is possible that the ion current could be estimated. Furthermore, it may be possible to use a single PMF to predict the current values under different voltages which could further save computational costs. One thing to keep in mind is that the ion occupancy probability is related to the free energy by an exponential relation. Thus, any uncertainty in the PMF (which are usually 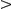
1 kcal/mol) will be amplified when determining the ion concentration to use in the NP equation, which could in turn result in a poor estimate of the ion current. Furthermore, there could be additional problems if multiple ions are resident in the channel which would be likely to require longer simulations to accurately sample all positions or to get multi-ion PMFs. Alternatively, Monte-Carlo approaches rather than dynamic ones may allow for more efficient sampling of the ion concentration as they can cover configurational space more efficiently [39], [40]. We suggest that further tests need to be carried out to see if a worthwhile means of combining MD or MC with NP to calculate channel currents can be devised. The PNP method might also be further improved, and our results here show that this ultimately requires the method to be able to determine the ion concentration accurately.

The systematic simulations and tests of the BD-NP method conducted here show that NP equation can be used to estimate ion currents provided they incorporate accurate ion concentrations and potentials. The accuracy could be probably further enhanced if a 3D NP equation is adopted. After verifying the validity of the NP theory in this way, the door is open to find efficient ways of combining microscopic and continuum approaches to predict ion channel currents. In this context, we believe that our study has provided a solid cornerstone for further effort in this direction.

## Supporting Information

Figure S1 **The sketch of 1D NP calculation.**
(TIF)

Figure S2 **The currents through the cylindrical channels when setting the dielectric constant of water to be 80.**
(TIF)

Figure S3
**The Na** 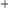 **currents for each sampling point calculated using the potential from Poisson's equation.** This example is from the real cylindrical channel of 6-Å radius.(TIF)

Figure S4
**Potential profiles calculated from Poisson's equation (BD) and our strategy (NP_Na** 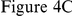 **and NP_Cl** 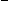 **), for a real cylindrical channel of 6-Å radius.**
(TIF)

Table S1 **The influence of grid spacing on the NP current.**
(TEX)

Table S2 **The influence of segment selection on NP current.**
(PDF)

Text S1 (PDF)
