# Supplementary figures and images for: Testing the Applicability of Nernst-Planck Theory in Ion Channels: Comparisons with Brownian Dynamics Simulations
Source: PLoS One. 2011 Jun 23;6(6):e21204. doi: 10.1371/journal.pone.0021204 (PMC3121742; doi:10.1371/journal.pone.0021204)

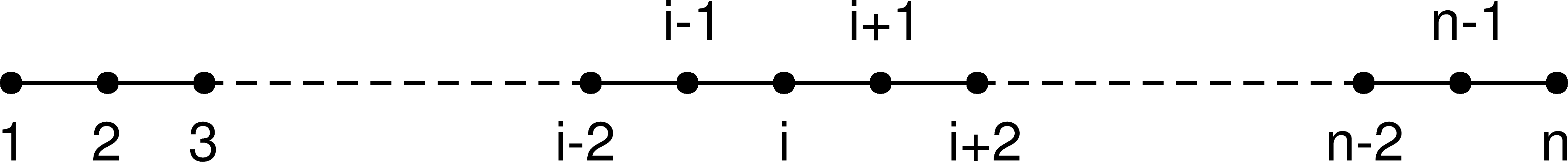

Supplement: Figure S1 — The sketch of 1D NP calculation. (TIF) [file pone.0021204.s001.tif]

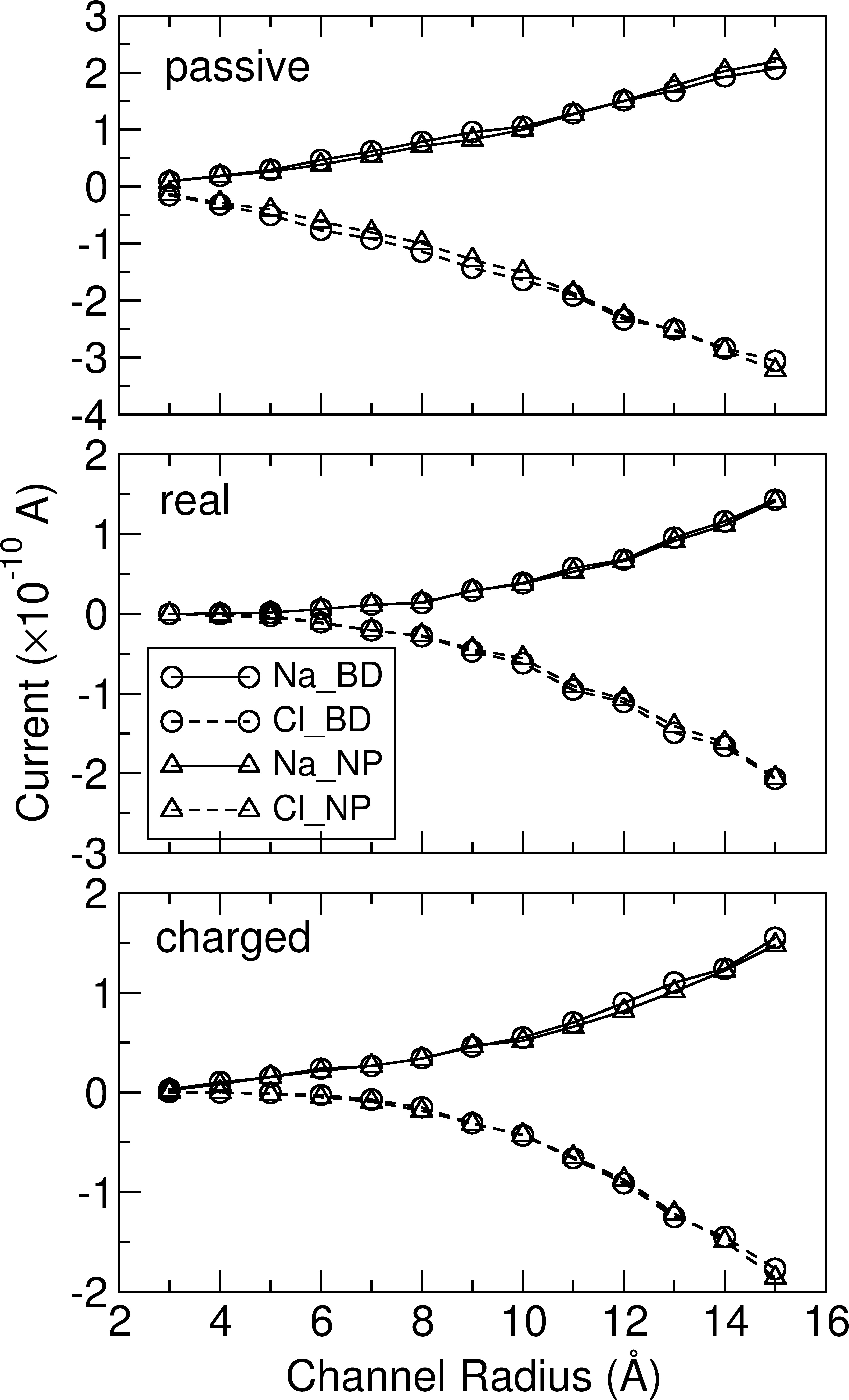

Supplement: Figure S2 — The currents through the cylindrical channels when setting the dielectric constant of water to be 80. (TIF) [file pone.0021204.s002.tif]

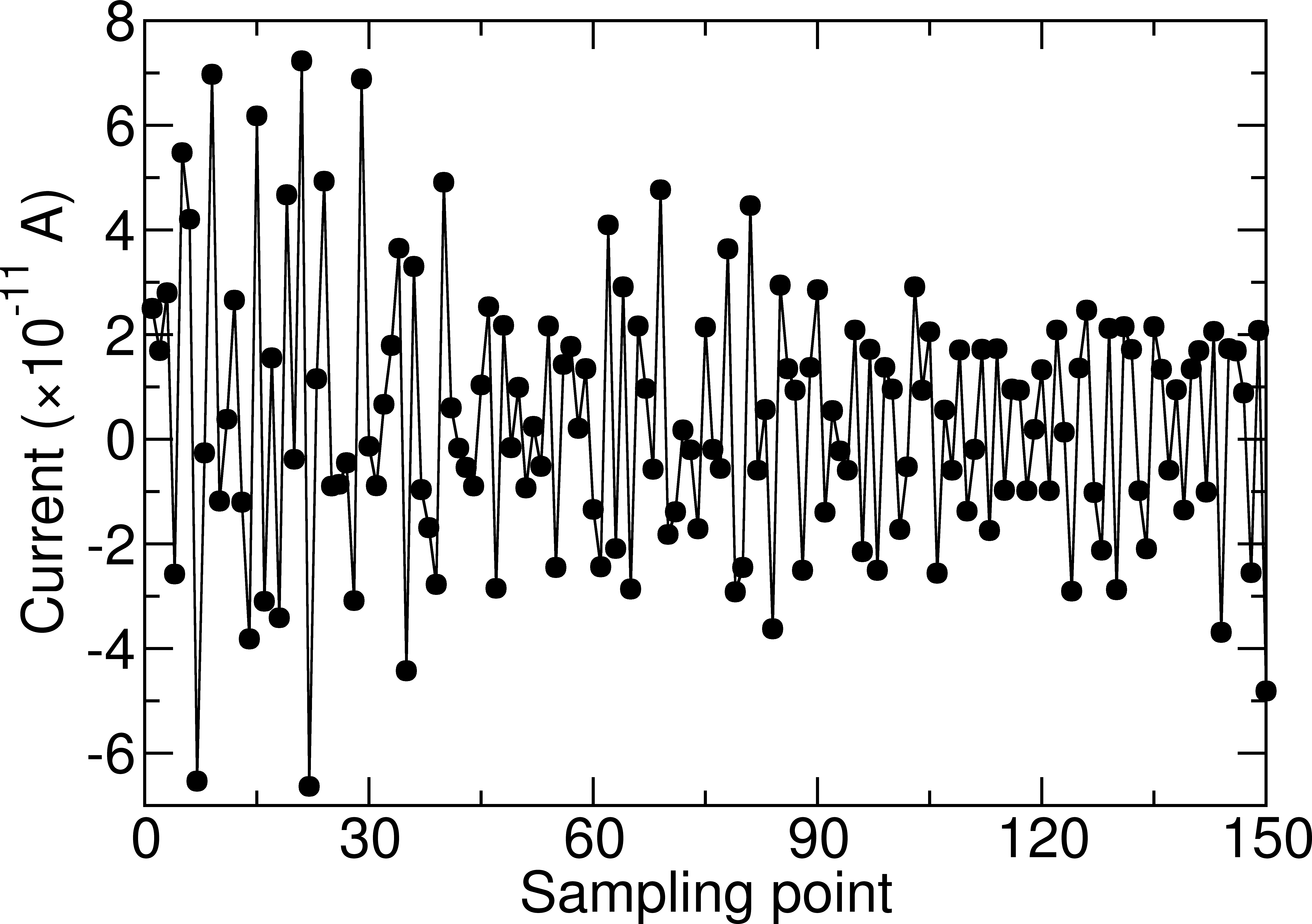

Supplement: Figure S3 — The Na currents for each sampling point calculated using the potential from Poisson's equation. This example is from the real cylindrical channel of 6-Å radius. (TIF) [file pone.0021204.s003.tif]

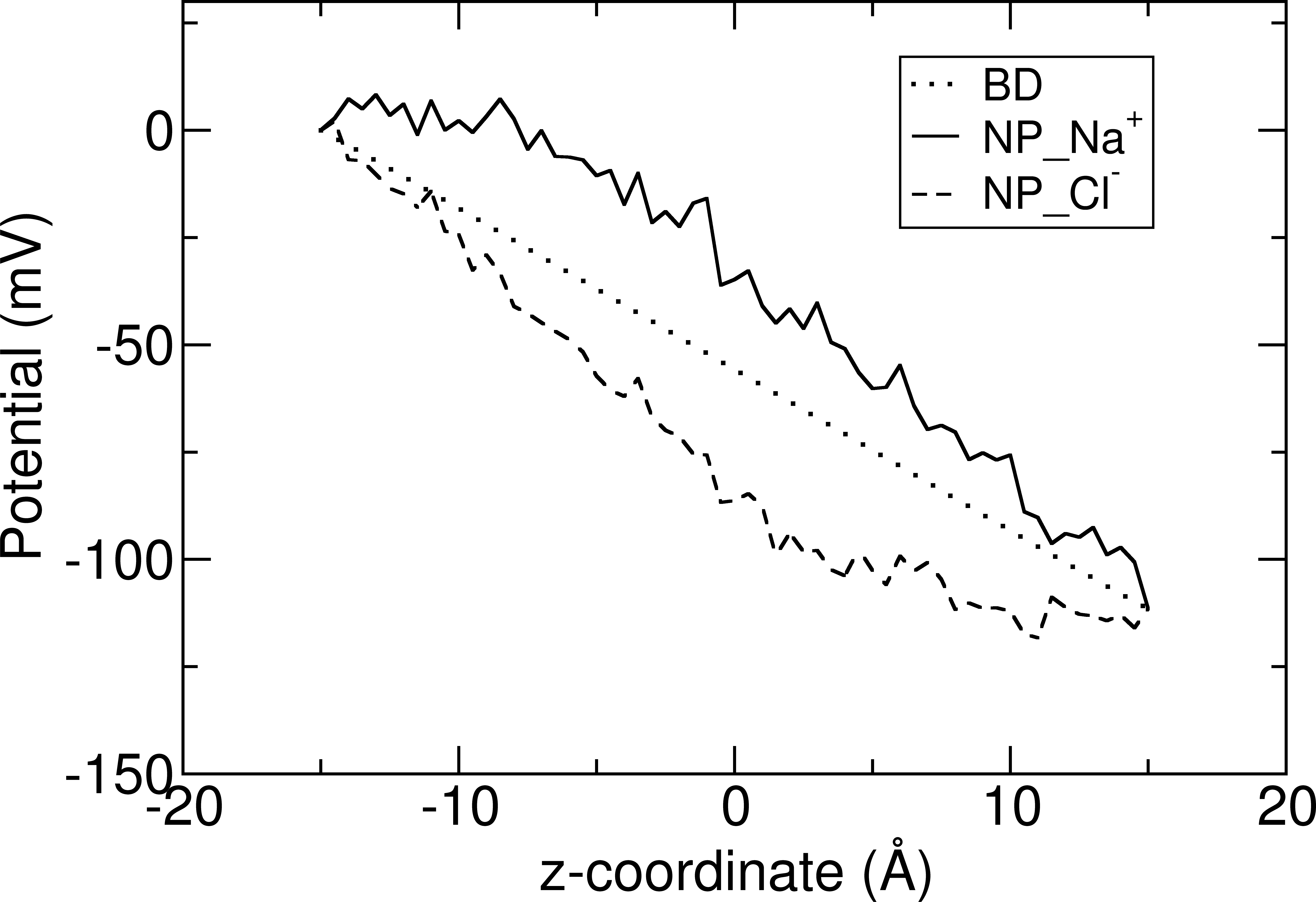

Supplement: Figure S4 — Potential profiles calculated from Poisson's equation (BD) and our strategy (NP_Na and NP_Cl ), for a real cylindrical channel of 6-Å radius. (TIF) [file pone.0021204.s004.tif]
