# Supplementary material for: Testing the Applicability of Nernst-Planck Theory in Ion Channels: Comparisons with Brownian Dynamics Simulations
Source: PLoS One. 2011 Jun 23;6(6):e21204. doi: 10.1371/journal.pone.0021204 (PMC3121742; doi:10.1371/journal.pone.0021204)
Supplement: Table S2 — The influence of segment selection on NP current. (PDF) [file pone.0021204.s006.pdf]

Table S2. The influence of segment selection on NP current (A). The BD results are 1.11×10^{-10} A and -5.69×10^{-11} A for Na^+ and Cl^- respectively.

segment	-10∼10	-15∼15	-20∼20	-30∼30
Na^+	1.12×10^{-10}	1.17×10^{-10}	1.19×10^{-10}	1.20×10^{-10}
Cl^-	-7.88×10^{-11}	-7.66×10^{-11}	-7.69×10^{-11}	-9.11×10^{-11}
