# Supplementary material for: Testing the Applicability of Nernst-Planck Theory in Ion Channels: Comparisons with Brownian Dynamics Simulations
Source: PLoS One. 2011 Jun 23;6(6):e21204. doi: 10.1371/journal.pone.0021204 (PMC3121742; doi:10.1371/journal.pone.0021204)
Supplement: Text S1 — (PDF) [file pone.0021204.s007.pdf]

# Testing the applicability of Nernst-Planck theory in ion channels: Comparisons with Brownian dynamics simulations (Supporting information)

Chen Song[1,2], Ben Corry[1,*]

**1 School of Biomedical, Biomolecular and Chemical Sciences, The University of Western Australia, Perth, Australia**

**2 Department of Theoretical and Computational Biophysics, Max Planck Institute for Biophysical Chemistry, Göttingen, Germany**

∗ **E-mail: ben.corry@uwa.edu.au**

## S1 Detailed implementation of the BD-NP method

To perform the NP calculations, three variables have to be determined. The first one is the diffusion coefficient, which was taken as the same values used in BD simulation in this study. The second one is the concentration for each of the ion species. And the third one is the potential. Here we describe how the latter two were determined from BD simulations for our 1D NP calculations.

### S1.1 The calculation of ion concentration from BD trajectory

The calculation of ion concentration is straightforward. As shown in figure S1, we consider 1D situation. We take a segment of the channel $z(1) \leq z \leq z(n)$ and divided it into $n-1$ small segments each of which has a length of $\delta$, so we have $n$ sampling points $(1, 2, \ldots, n)$. Assuming $i$ is one arbitrary sampling point, then the concentration of the ion species $v$ at point $i$, $C_v(i)$, is calculated from the BD trajectories as follows:

$$C_v(i) = \frac{N_v(i)}{(\pi \times R(i)^2 \times \delta) \times n_f}, \tag{S1}$$

$N_v(i)$ is the number of ions $v$ that locating at $z(i) - \frac{\delta}{2} \leq z \leq z(i) + \frac{\delta}{2}$ in the entire production trajectory, $R(i)$ is the radius of the channel at the sampling point $i$, and $n_f$ is the total amount of the frames in the production trajectories. By this calculation, the concentration of each ion species at all the $n$ sampling points are known.

## S1.2 The calculation of potential

In this study we calculated the potential at each sampling point using two steps. In the first we solved Poisson's equation to determine the total drop in potential, $V$, between the two ends of the segment used for the calculation, i.e., between sampling points 1 and $n$. For this calculation, the external applied electric field, the dielectric boundary, and the fixed charges on the channel were all considered. While the mobile ions were not. Then, we used the stationary NP equation to determine the potential at the intermediate points by utilising the values of the concentration at each point.

Assuming the potential at the first sampling point

$$\Phi(1) = 0, \tag{S2}$$

then the potential at the last sampling point

$$\Phi(n) = V. \tag{S3}$$

To fulfil the stationary NP equation, we have

$$\nabla \mathbf{J}_v = 0 \tag{S4}$$

which in the 1D case is

$$J_v(i) = J_v(i+1) \tag{S5}$$

Substituting the 1D NP equation

$$J_v = -D_v(\frac{n_v(i+1) - n_v(i)}{\delta} + \frac{z_v e n_v(i)}{kT}\frac{\Phi(i+1) - \Phi(i)}{\delta}), \tag{S6}$$

into the equation S5, and noting that $n_v(i) = 10^3 N_A C_v(i)$, one gets

$$
\begin{aligned}
&-C_v(i)\Phi_v(i) + [C_v(i) + C_v(i+1)]\Phi_v(i+1) - C_v(i+1)\Phi_v(i+2) \\
&\qquad = [C_v(i+2) - 2 \times C_v(i+1) + C_v(i)] \times \frac{kT}{z_v e}
\end{aligned} \tag{S7}
$$

If we consider the channel cross section area

$$
\begin{aligned}
&-C_v(i)S(i)\Phi_v(i) + [C_v(i)S(i) + C_v(i+1)S(i+1)]\Phi_v(i+1) - C_v(i+1)S(i+1)\Phi_v(i+2) \\
&\qquad = [C_v(i+2)S(i+1) - C_v(i+1)S(i+1) - C_v(i+1)S(i) + C_v(i)S(i)] \times \frac{kT}{z_v e}
\end{aligned} \tag{S8}
$$

where $C_v(i)$ is the concentration of the ion species $v$, $S(i)$ is the channel cross section area, and $\Phi_v(i)$ is the potential at the $i$th sampling point respectively.

Equation S8 gives us $n-2$ equations ($i = 1, 2, \ldots, n-2$). Combining them with the equations S2 and S3, we get a system of $n$ equations. As the ion concentration $C_v(i)$, the channel cross section area $S(i)$, and the potential difference $V$ are already known, the system of equations can be solved to get the potential for each ion species $\Phi_v(i)$. By using this strategy, it is guaranteed that the current through each sampling section is the same and the choice of segment on which the NP calculation is performed does not affect the result.

## S1.3 The calculation of ion current

Once we have obtained the ion concentration and potential at each sampling point, the calculation of ion current can be done by:

$$
I(i) = J(i) \times S(i) = -D_v \times \left(\frac{n_v(i+1) - n_v(i)}{\delta} + \frac{z_v e n_v(i)}{kT}\frac{\Phi(i+1) - \Phi(i)}{\delta}\right) \times S(i) \tag{S9}
$$

where $n_v(i) = 10^3 N_A C_v(i)$.

### S1.4 The selection of the grid spacing

In our calculations, the grid spacing $\delta$ was set to 0.5 Å, as our tests show that a value of $0.5 \sim 1.0$ gives the most stable prediction about the ion current. Using smaller values requires longer simulations to achieve the same level of sampling, while coarser grids can begin to compromise the results. The table S1 shows an example of how the grid spacing influences the calculated current found in the case of the 'real catenary channel'.

**Table S1.** The influence of grid spacing $\delta$ (in Å) on NP current ($\times 10^{-11}$ A). The BD results are 2.39 and -3.89 for $Na^+$ and $Cl^-$ respectively.

| $\delta$ | 0.1 | 0.2 | 0.3 | 0.4 | 0.5 | 1.0 | 1.5 | 2.0 | 3.0 | 4.0 | 5.0 |
|---|---|---|---|---|---|---|---|---|---|---|---|
| $Na^+$ | 1.09 | 2.17 | 2.45 | 2.48 | 2.56 | 2.56 | 2.54 | 2.52 | 2.48 | 2.53 | 2.39 |
| $Cl^-$ | -7.00 | -5.12 | -4.64 | -4.62 | -4.55 | -4.54 | -4.62 | -4.71 | -4.92 | -5.15 | -5.38 |

### S1.5 The influence of the choice of the segment to perform the NP calculation

In one of our catenary channel calculations (cylindrical part has a radius of 8 Å and spans from z=-7.5 Å to z=7.5 Å. The widest part has a maximum radius of 12 Å at z=22.5 Å) , we studied if selecting different segments to perform the NP equation would affect the accuracy. The results are shown in table S2.

**Table S2.** The influence of segment selection on NP current (A). The BD results are $1.11\times10^{-10}$ A and $-5.69\times10^{-11}$ A for $Na^+$ and $Cl^-$ respectively.

| segment | -10∼10 | -15∼15 | -20∼20 | -30∼30 |
|---|---|---|---|---|
| $Na^+$ | $1.12 \times10^{-10}$ | $1.17 \times10^{-10}$ | $1.19 \times10^{-10}$ | $1.20 \times10^{-10}$ |
| $Cl^-$ | $-7.88 \times10^{-11}$ | $-7.66 \times10^{-11}$ | $-7.69 \times10^{-11}$ | $-9.11 \times10^{-11}$ |

As can be seen, only after including the ends of the channel for the calculation, there is a relatively larger deviation, especially for $Cl^-$. Therefore, we suggest when doing such kind of calculation, one should try to take part of the channel and avoid including the ends of the channel or water reservoir.

## S2 The influence of different dielectric constants

In the paper, all the calculations were performed with the dielectric constant of water set to 60.0. To help see the influence of this choice of dielectric constant on our results, we also performed similar simulations

for passive, real and charged cylindrical channels with the dielectric constant of water to be 80 and all the other parameters be the same. The results are shown in figure S2.

Increasing the dielectric constant of water increases the ion conductance through the channel. However, as can be see in the figure S2, the BD-NP results still match well with the BD results indicating that the choice of the dielectric constant does not have a significant affect on the accuracy of the NP calculations.

# S3 Alternative methods for calculating the electrostatic potential

One might ask why we bothered to calculate the potential from the ion concentration via the NP equation instead of using the potential calculated from Poisson's equation directly. In fact, we have tried this direct method but encountered some practical issues. When employing this strategy we find that the values we determine for the current at each sampling point fluctuate significantly throughout the channel as shown in figure S3. In reality this does not make sense as the current in a single channel should be the same at every cross section. This fluctuation arises due to the sensitivity of the current calculated using the NP equation to the specific value of the concentration and potential at that point. Thus, any small deviations in average concentration of potential arising from insufficient sampling are amplified.

It should be noted that the average value found from the fluctuating currents as shown in figure S3 still do make sense and match well with the BD results for the cylindrical channels. For instance, the average value calculated for the above example using the middle 90 sampling points is 2.42 $\times 10^{-12}$ A, which is very close to the BD result $2.06\times 10^{-12}$ A. (The result using the BD-NP approach employed in the manuscript is 2.06 $\times 10^{-12}$ A.) But for more complex channels, this method fails and can not give stable current values which are dependent on the choice of sampling points. The fact that the results become dependent on the choice of the sampling points in this direct method creates a significant drawback.

## S3.1 Potential problem in our strategy

There is one problem about our strategy of calculating the potential from ion concentration: the potential profiles calculated for different ion species do not match with each other.

As shown in figure S4, for the same channel, the potentials calculated from [$Na^+$] and [$Cl^-$] do not match with each other, and both of them deviate from the electrostatic calculation results using Poisson's equation. This is a drawback of our strategy and can not be avoided in order to satisfy mass continuity (stationary NP equation S5).

Having examined both of the above two methods for calculating the potential, we believe that the one employed in the manuscript that uses the NP equation to derive the potential using mass continuity works much better. Although the potentials calculated from different ion species do not match with each other, our results show that it can predict a stable flux that matches well with the BD results. Having said this, it is possible that the method of determining the electrostatic potential could be improved in the future.
